# Development and Assessment of a Novel Predictive Nomogram to Predict the Risk of Secondary CR-GNB Bloodstream Infections among CR-GNB Carriers in the Gastroenterology Department: A Retrospective Case–Control Study

**DOI:** 10.3390/jcm12030804

**Published:** 2023-01-19

**Authors:** Hongchen Zhang, Shanshan Hu, Lingyun Li, Hangbin Jin, Jianfeng Yang, Hongzhang Shen, Xiaofeng Zhang

**Affiliations:** 1 The Fourth School of Clinical Medicine, Zhejiang Chinese Medical University, Hangzhou 310003, China; 2 The Department of Gastroenterology, Affiliated Hangzhou First People’s Hospital, Zhejiang University School of Medicine, Hangzhou 310006, China; 3 Key Laboratory of Integrated Traditional Chinese and Western Medicine for Biliary and Pancreatic Diseases of Zhejiang Province, Hangzhou 310000, China; 4 Hangzhou Institute of Digestive Disease, Hangzhou 310000, China

**Keywords:** carbapenem-resistant Gram-negative bacteria, blood stream infection, gastroenterology department, predictive nomogram, empirical antibiotic therapy

## Abstract

Background: With the number of critically ill patients increasing in gastroenterology departments (GEDs), infections associated with Carbapenem-resistant Gram-negative bacteria (CR-GNB) are of great concern in GED. However, no CR-GNB bloodstream infection (BSI) risk prediction model has been established for GED patients. Almost universally, CR-GNB colonization precedes or occurs concurrently with CR-GNB BSI. The objective of this study was to develop a nomogram that could predict the risk of acquiring secondary CR-GNB BSI in GED patients who are carriers of CR-GNB. Methods: We conducted a single-center retrospective case–control study from January 2020 to March 2022. Univariate and multivariable logistic regression analysis was used to identify independent risk factors of secondary CR-GNB bloodstream infections among CR-GNB carriers in the gastroenterology department. A nomogram was constructed according to a multivariable regression model. Various aspects of the established predicting nomogram were evaluated, including discrimination, calibration, and clinical utility. We assessed internal validation using bootstrapping. Results: The prediction nomogram includes the following predictors: high ECOG PS, severe acute pancreatitis, diabetes mellitus, neutropenia, a long stay in hospital, and parenteral nutrition. The model demonstrated good discrimination and good calibration. Conclusions: With an estimate of individual risk using the nomogram developed in this study, clinicians and nurses can identify patients with a high risk of secondary CR-GNB BSI early.

## 1. Introduction

Carbapenem-resistant Gram-negative-bacteria (CR-GNB), namely, carbapenem-resistant Enterobacteriaceae (CRE) (for example, *Klebsiella pneumoniae* and *Escherichia coli*), *Acinetobacter baumannii* (CRAB) and *Pseudomonas aeruginosa* (CRPsA), are important multidrug-resistant bacteria around the world that can induce serious infections [1]. Most hospital-acquired infections (HAI) are caused by these infections, which are related to a higher mortality rate and a longer hospital stay [2]. China has also faced challenges due to CR-GNB infection. Based the data from 2021 China Antimicrobial Surveillance Network report, the resistance rates of *Klebsiella pneumoniae*, *Pseudomonas aeruginosa*, and *Acinetobacter baumannii* have risen above 23%, 20%, and 73%, respectively [3]. Globally, the WHO and USCDCP have both determined the CR-GNB risk level as the highest and recommended active and effective preventative measures against CR-GNB infection to ensure the safety of patients.

To reduce the spread of CR-GNB, it is important to detect CR-GNB carriers as early as possible and take other infection control measures, such as preemptive isolation and cohorting. Almost universally, CR-GNB colonization precedes or occurs concurrently with CR-GNB infection [4]. Early detection of CR-GNB colonization can therefore assist in identifying patients with the highest risk of CR-GNB infection in the future. Meta-analysis has demonstrated that CR-GNB carrier patients were 16.5% more likely to become infected [5]. In recent studies, it has been shown that approximately 11% to 30% of patients became infected after colonization with CR-GNB and that the endemicity of CR-GNB as well as IPC strategies influenced infection rates [6].

CR-GNB can be treated with polymyxin B or ceftazidime-avibactam, but there have also been other measures developed [7]. It is currently recommended to use early empirical antibiotic treatments for severe infections, but guidelines tend to be cautious when it comes to the optimal antibiotic timing in cases of CR-GNB infection, since there are few effective antibiotics, and “old” antibiotics are potentially ineffective [8]. After CR-GNB infection, antibiotics are usually prescribed after the positive yield of blood culture, which may delay treatment for at least 2–3 days. According to several recent studies, using sensitive antibiotics earlier may reduce CR-GNB bloodstream infection mortality [8,9,10]. Nevertheless, indiscriminate use of antibiotics can lead to increased resistance to antibiotics. It is, therefore, possible to construct a risk prediction model for secondary CR-GNB blood stream infection (BSI) in CR-GNB carriers once risk factors of CR-GNB infection are clarified. In addition, high-risk-guided empirical anti-CR-GNB antibiotic treatment may be more appropriate for CR-GNB-colonized patients when BSI symptoms occur.

As technology develops in the field of digestive endoscopy, more critically ill patients with malignant or infectious diseases of the biliopancreatic system and major gastrointestinal tract surgery history have been admitted in the gastroenterology department (GED). There is usually a poor prognosis and high mortality rate associated with CR-GNB BSI in these patients [11]. Recently, the incidence of CR-GNB BSI among GED patients has increased dramatically, posing a serious health threat [12]. Since almost all studies have involved a case mix of ICU and transplant ward patients, or few GED patients, there is a lack of data on the GED population [13,14,15]. The risk of CR-GNB BSI among CR-GNB-carrier patients in GED has not yet been established. Our analysis for the first time took into account the unique characteristics of the GED patient population, which included a range of common digestive disorders and endoscopic interventional procedures. In our study, secondary CR-GNB BSI risk factors among carriers were explored, and a nomogram was developed to assess the risk of secondary CR-GNB BSI among carriers.

## 2. Materials and Methods

### 2.1. Study Design and Setting

This was a retrospective observational case–control study conducted in the department of gastroenterology of Affiliated Hangzhou First People’s Hospital of Zhejiang University School of Medicine, a tertiary-first-class academic hospital, with 150 beds in 3 regular floor wards of GED in Hangzhou, China. In this study, the study period lasted from 1 January 2020 to 31 March 2022. As the region is known to be an endemic area for cases of CR-GNB, a universal rectal swab performed at admission in GED was conducted as part of the hospital’s infection control policy during this period and re-examined regularly. CR-GNB carriage status was monitored actively and the CR-GNB carriers were isolated individually. Data from 1 January 2020 to 31 March 2022were collected. The patients with a positive result from the CR-GNB screening test in GED were enrolled in our study. Positive results on the screening test for CR-GNB without invasive infection were defined as CR-GNB intestinal colonization. CR-GNB BSI diagnosis was determined by two senior attending physicians independently. Infections involving CR-GNB strains isolated from blood cultures and exhibiting clinical manifestations were defined as CR-GNB BSI. Patients under 18 years old, patients with incomplete clinical records and patients with inconsistent screening results were excluded. Because the study focused on risk factors of nosocomial infections, cases of CR-GNB BSI occurring before or within 48 h of admission were excluded to ensure that the cases were nosocomial infections and to avoid the influence of confounding factors of community infection. Individuals with CR-GNB BSI subsequent to CR-GNB intestinal colonization were included in the case group. CR-GNB-carrier individuals without secondary CR-GNB BSI were included in the control group. It is possible that estimates may be biased due to previous differences between case and control group characteristics. Propensity score matching (PSM) techniques were used to reduce this bias. As a result, the case and control groups were matched 1:1 using PSM based on received GED regular floor wards. A total of 90 patients with CR-GNB BSI subsequent to CR-GNB intestinal colonization and 90 CR-GNB carrier patients without secondary CR-GNB BSI were enrolled in the study. This study aimed to identify risk factors associated with secondary CR-GNB BSIs subsequent to CR-GNB colonization in GED patients. Ethics approval was obtained from the ethics committee of Affiliated Hangzhou First People’s Hospital of Zhejiang University School of Medicine before the study was conducted (Ethical approval number: ZN2022062, 11 May 2022). The study was conducted in accordance with GCP and the Declaration of Helsinki.

### 2.2. Microbiology

In order to screen for CR-GNB, rectal swabs were directly inoculated onto chromogenic agar plates containing carbapenem (CHROMagar, Paris, France). A MALDI-TOF mass spectrometer was used to identify all the isolated bacteria (Bruker Daltonics, Billerica, MA, USA) [16]. We measured the sensitivity of imipenem and meropenem using Kirby-Bauer’s method [17]. The results were interpreted according to M100-ED30 breakpoints established by the Clinical and Laboratory Standards Institute (CLSI).

### 2.3. Variables and Definitions

Electronic medical records were used to collect the data retrospectively. An analysis of all variables potentially associated with secondary CR-GNB BSI was conducted. These variables include general information (age, gender, wards, CR-GNB isolate, Eastern Cooperative Oncology Group score [18]), past history (long-term stay in a healthcare facility within 1 year, ICU admission history within 1 year, blood-stream infection history within 1 year, transfer from another healthcare facility and gastrointestinal divert history), underlying conditions (gastrointestinal bleeding, inflammatory bowel disease, severe acute pancreatitis, acute cholangitis, cirrhosis, gastrointestinal obstruction, gastrointestinal cancer, diabetes, cardial vascular disease, cerebral vascular disease, pulmonary disease, uremia, neutropenia), endoscopy interventions and other interventions after survey (gastroscopy, colonoscopy, ercp, enbd, small bowel feeding tube insertion, small bowel decompression tube insertion, colon decompression tube insertion, deep venous catheter insertion, dialysis, cholecystostomy, ptcd and parenteral nutrition), drugs and antibiotic exposure after survey (chemotherapy, long-term steroid usage longer than 5 days, β-lactam-β-lactamase inhibitor, cephalosporins, quinolone, carbapenem and poly antibiotics) and admission duration (length of stay from CR-GNB screening to outcome: occurrence of CR-GNB BSI or discharge; long-term stay in hospital, which is defined as a length of stay from CR-GNB screening to outcome of more than 13 days). Important definitions were depicted as follows. Severe acute pancreatitis is diagnosed when pancreatitis patients have persistent organ failure which does not resolve within 48 h [19]. Acute cholangitis is diagnosed by the identification of the clinical manifestations of Charcot’s triad, including fever and/or chills, abdominal pain and jaundice [20]. Neutropenia is defined as an absolute neutrophil count (ANC) of less than 1500/mcL.

### 2.4. Statistical Analysis

Statistics relating to demographics, disease, and treatment are expressed as counts (%). Using the R programming language (Version 3.1.1; https://www.R-project.org (accessed on 12 January 2023)), we performed the statistical analysis. To select optimal predictive features in risk factors from secondary CR-GNB BSI carrier patients, we used the least absolute shrinkage and selection operator (LASSO) method, suitable for reducing high-dimensional data [21]. An analysis of the LASSO regression model was conducted to identify features with nonzero coefficients. After that, the chosen features were incorporated into a multivariable logistic regression model to build a prediction model [22,23]. The features were considered as an odds ratio (OR) with a 95% confidence interval (CI) and as the *p*-value. A two-sided significance level was used for all the statistical tests. Statistically significant sociodemographic variables were included in the model, as well as variables pertaining to disease and treatment characteristics. By using the cohort, all potential predictors were applied to create a predicting model for secondary CR-GNB BSI risk [24]. Using calibration curves, we next evaluated the calibration of the BSI risk nomogram. The presence of a significant test statistic implies that the model is not calibrated perfectly. A measurement of Harrell’s C-index was performed to quantify the discrimination performance of the secondary CR-GNB BSI risk nomogram. To calculate a relatively corrected C-index, a bootstrapping validation (1000 resamples) was performed on the BSI risk nomogram [25]. Based on the numerical results from a decision curve analysis, the clinical usefulness of the secondary CR-GNB BSI risk nomogram was evaluated at a variety of threshold probabilities by quantifying the net benefits in the GED patient cohort [26]. To determine the net benefit, we subtracted the proportion of false positives from the proportion of true positives and assessed the relative harm of not intervening versus the negative outcomes of unnecessary intervention.

## 3. Results

### 3.1. Characteristics of the Included Patients

Overall, 1100 patients were initially qualified and included in this study; 90 patients (8.2%) developed secondary CR-GNB BSI after admission. After propensity score matching, 180 patients were analyzed in our study. Baseline characteristics are shown in Table 1. Both case and control group patients were matched in terms of being on which regular floor wards. There was no significant difference in age and gender between both groups. In terms of past history, such as a previous long-term stay in a healthcare facility, ICU admission history and blood stream infection history within 1 year, there was no significant difference between groups. When comparing CR-GNB isolates, a higher proportion of CRKP was found in the case group. A significant difference was found between case and control groups in terms of CR-GNB isolates (*p* < 0.01), ECOG performance status (PS) (*p* < 0.001), severe acute pancreatitis (11% and 33%, respectively, *p* < 0.001), diabetes mellitus (22% and 37%, respectively, *p* < 0.05), neutropenia (7% and 27%, respectively, *p* < 0.001), long-term stay in hospital (43% and 56%, respectively, *p* < 0.001) and parenteral nutrition (11% and 36%, respectively, *p* < 0.001). Interestingly, the proportion of small intestinal feeding tube usage was higher in control group patients (16% and 3%, respectively, *p* < 0.005).

### 3.2. An Analysis of Multivariate and Univariate Logistic Regression to Identify the Risk Factors for Secondary CR-GNB BSI from Colonization

To identify the risk factors that exhibited statistical differences between both groups, univariate logistic regression analyses were conducted. It was observed that ECOG PS, severe acute pancreatitis, diabetes mellitus, neutropenia, long-term stay in hospital, *Klebsiella pnenmoniae* and parenteral nutrition are the risk factors affecting secondary CR-GNB BSI, as shown in Table 2.

In addition to univariate logistic regression, multivariate logistic regression was conducted on the risk factors determined by univariate logistic regression. By adjusting confounders, we identified ECOG PS (odds ratio (OR) 5.6, 95% confidence interval (CI) 2.96–10.90, *p* < 0.001), severe acute pancreatitis (OR 6.32, 95% CI 2.02–19.81, *p* < 0.01), diabetes mellitus (OR 4.02, 95% CI 1.40–11.50, *p* < 0.05), neutropenia (OR 4.77, 95% CI 1.40–15.76, *p* < 0.05), long-term stay in hospital (OR 5.32, 95% CI 2.25–12.54, *p* < 0.001) and parenteral nutrition above 3 days (OR 9.01, 95% CI 2.77–29.36, *p* < 0.001) to be independent risk factors affecting the risk of secondary CR-GNB BSI from colonization, as shown in Table 2.

### 3.3. Feature Selection

**Figure 1:**
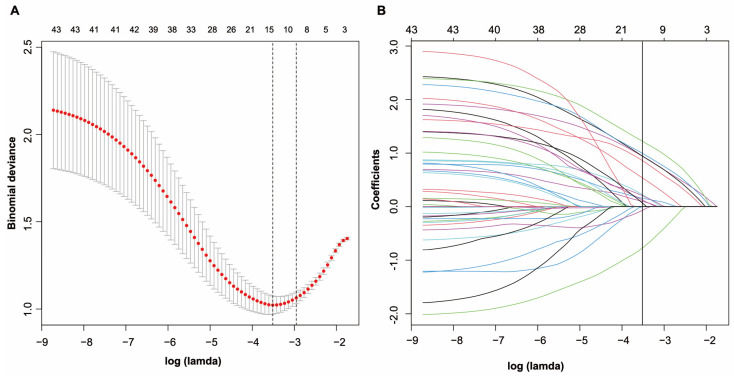
Selection of demographic and clinical features using LASSO binary logistic regression. (**A**) In the LASSO model, an optimal parameter (lambda) was selected using five-fold cross-validation based on minimum criteria. An image of the partial likelihood deviance (binomial deviance) curve was plotted against log (lambda). Based on the minimum criteria as well as 1 SE from the minimum criteria, dots were drawn on the vertical axis corresponding to the optimal values. (**B**) LASSO coefficient profiles of the 43 features. A coefficient profile plot was produced against the log (lambda) sequence. A vertical line was drawn at the value selected using fivefold cross-validation, where the optimal lambda resulted in fifteen features with nonzero coefficients. Abbreviations: LASSO (least absolute shrinkage and selection operator); SE (standard error).

As shown in Figure 1A,B, a LASSO regression analysis of 180 patients in the cohort revealed that 15 potential predictors identified from 43 features were with nonzero coefficients (~4:1 ratio). These features included *Klebsiella pneumonia*, high ECOG PS, severe acute pancreatitis, diabetes, parenteral nutrition, long-term stay in hospital, chemotherapy usage after survey, long-term stay in healthcare facility within 1 year, neutropenia, acute cholangitis, colonoscopy, small bowel feeding tube insertion, cholecystostomy and carbapenem usage after survey.

### 3.4. Model Development for Individualized Prediction

**Figure 2:**
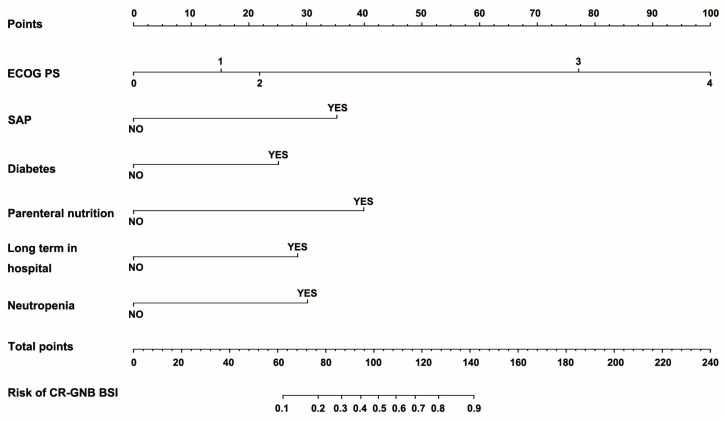
Nomogram for predicting the probability of secondary CR-GNB BSI for carrier patients in GED. Abbreviations: ECOG (Eastern cooperative oncology group score); SAP (severe acute pancreatitis); CR-GNB (carbapenem–resistant Gram–negative bacteria); BSI (blood stream infection).

**Figure 3:**
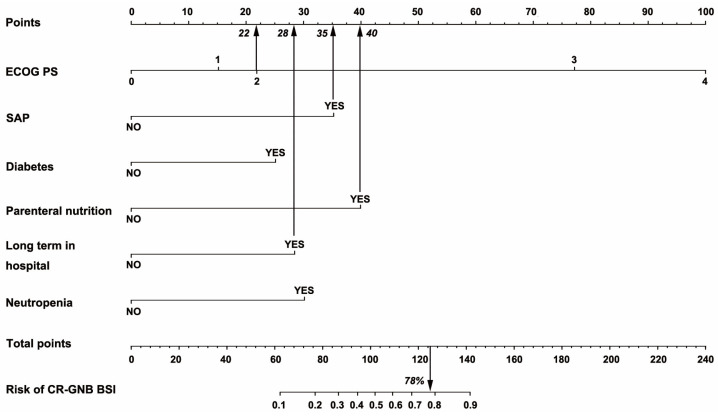
The example of how the predictive nomogram can be used. Abbreviations: ECOG (eastern cooperative oncology group score); SAP (severe acute pancreatitis); CR-GNB (carbapenem resistant Gram negative bacteria); BSI (blood stream infection).

**Figure 4:**
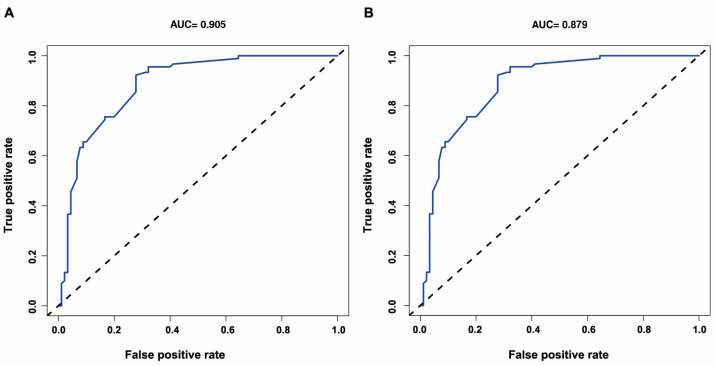
ROC curves validating the discrimination power of the nomogram ((**A**) training; (**B**) bootstrapping). Abbreviations: AUC (area under the curve).

**Figure 5:**
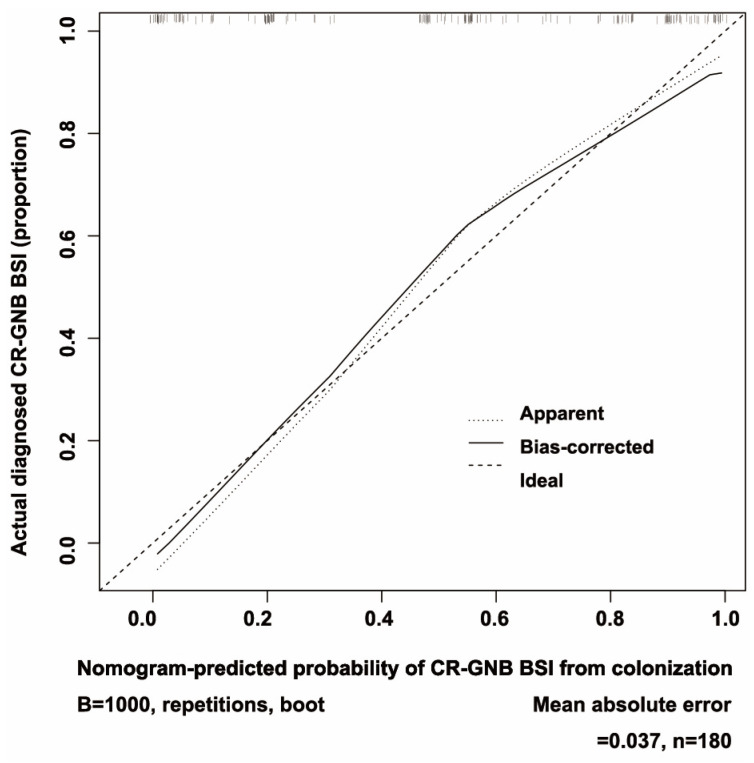
An assessment of the calibration curves for secondary CR-GNB BSI risk nomogram prediction. The x-axis shows the predicted risk of secondary CR-GNB BSI. The y-axis shows the actual secondary CR-GNB BSIs that have been diagnosed. The diagonal dotted line in the diagram represents the prediction of the ideal model. The solid line represents nomogram performance, with a closer fit to the diagonal dotted line representing a better prediction. Abbreviations: CR-GNB (carbapenem-resistant Gram-negative bacteria); BSI (blood stream infection).

Nomograms (Figure 2) were developed by incorporating the identified independent predictors. Figure 3 shows an example of how the nomogram can be used in terms of the nomogram’s apparent performance for detecting CR-GNB carrier’s risk of secondary CR-GNB BSI. The prediction nomogram’s C-index was 0.905, and bootstrapping validation demonstrated the model’s good discrimination, which was confirmed by the C-index of 0.879 for the cohort (Figure 4). As shown in Figure 5, the calibration curve for the predictive nomogram performed well in this cohort for predicting secondary CR-GNB BSI risk. According to the CR-GNB BSI risk prediction nomogram, apparent performance is a good indicator of prediction accuracy.

### 3.5. Clinical Use

**Figure 6:**
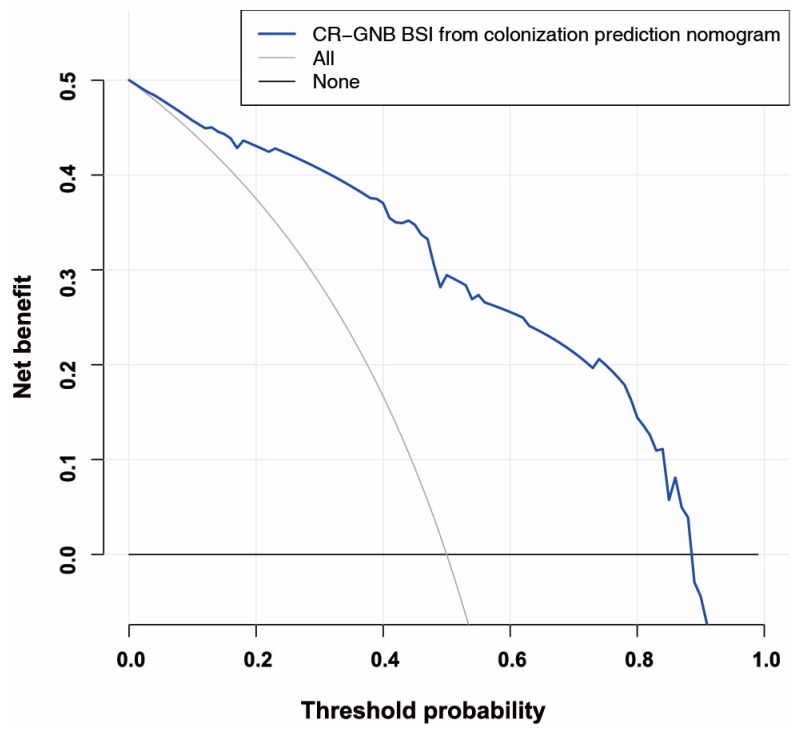
The decision curve of the secondary CR-GNB BSI risk nomogram is analyzed. Net benefit is measured on the y-axis. The dotted line represents the BSI risk nomogram for CR-GNB. In this graph, the thin solid line indicates that all patients have secondary CR-GNB BSI. The thick solid line represents the assumption that no secondary CR-GNB BSI has occurred in any patient. Based on the decision curve, if the patient’s threshold probability was between 1% and 95%, using this model to predict secondary CR-GNB BSI would result in a greater net benefit for the patient. Abbreviations: CR-GNB (carbapenem-resistant Gram-negative bacteria); BSI (blood stream infection).

Figure 6 illustrates the decision curve analysis for the secondary CR-GNB BSI nomogram. Based on the decision curve, if the patient’s threshold probability was between 1% and 90%, using this model to predict secondary CR-GNB BSI for carrier patients would result in a greater net benefit for the patient.

## 4. Discussion

In this study, we identified the risk factors of secondary CR-GNB BSI from colonization among carrier patients in GED, designed a nomogram to determine secondary CR-GNB BSI risk, and evaluated the performance of the nomogram using internal validation. In the training set and subsets, the nomogram based on these six factors showed good calibration, discrimination, and clinical utility. As a result of the nomogram constructed in this study, healthcare workers can calculate CR-GNB BSI risk for the carrier individual in GED with ease. The nomogram is therefore expected to contribute to the early detection of high-risk secondary CR-GNB BSI patients and rapid decision making, whether empirical anti-CR-GNB antibiotic treatment is needed or not.

In our study, we investigated the risk factors for acquiring secondary CR-GNB BSI among carrier patients in GED. Our findings revealed that high ECOG PS was associated with higher secondary BSI risk with CR-GNB. There are many factors that may affect ECOG PS, such as the patient’s age, the burden of their illnesses, the stage of their cancer, and the side effects of their chemotherapy treatment [18]. Patients with a poor ECOG PS and limited functional capacity always suffer from compromised immune function, which might result in more vulnerability to CR-GNB BSI [27]. It was demonstrated in our study that poor ECOG PS was an independent risk factor for secondary CR-GNB BSI for carrier patients of GED, whereas malignant disorder or chemotherapy treatment were not. It is well-acknowledged that, as a result of immunocompromised conditions, the body’s normal defense mechanisms are compromised, which might predispose these patients to life-threatening infections such as CR-GNB sepsis that may not otherwise occur [28]. In GED patients, there are a variety of host abnormalities associated with an impaired immune system. These disorders include but are not limited to diabetes, chronic liver disease and disease of the cardiopulmonary system [29,30]. Therefore, we took these factors into account when analyzing secondary CR-GNB BSI risk factors. Our study demonstrated that diabetes and neutropenia are the most concerning patient risk factors for secondary CR-GNB BSI for carriers in GED. Considering the specificity of the patient population in GED, a range of common digestive disorders and commonly performed endoscopy interventions were included in the univariate and multivariate analyses. It is revealed that severe acute pancreatitis was found to be closely associated with secondary CR-GNB BSI among carrier patients. It is speculated that, during acute inflammation, the pancreas is more susceptible to bacterial infection. On the other hand, secondary intestinal mucosal barrier deficiency allows CR-GNB organisms to translocate into the systemic circulation and abdominal organs, which results in supervening sepsis and critical complications.

Furthermore, it is interesting to note that patients receiving parenteral nutrition for more than 3 days are susceptible to CR-GNB BSI. Enteral nutrition through the insertion of small intestine feeding tubes; however, is the protective factor against secondary CR-GNB BSI. In a previous study, enteral nutrition was significantly associated with fewer infectious complications than parenteral nutrition in pancreatitis patients [31]. It is speculated that, by preserving the barrier function of the intestine, enteral nutrition can reduce bacterial translocation. Our data also demonstrated that a prolonged length of stay of more than 13 days was also an independent risk factor for secondary CR-GNB BSI among carriers.

Currently, secondary CR-GNB BSI is strongly associated with several risk factors. To date, no scoring system has been established to predict secondary CR-GNB BSI from colonization in GED. Taking advantage of our data, we established a predictive nomogram to assess the CR-GNB BSI risk among carrier patients in GED, which performed well. By utilizing the developed nomogram, clinicians can assess each patient’s risk of acquiring a secondary CR-GNB BSI. According to growing evidence, early prescription of empirical antibiotics for severe sepsis patients may reduce mortality. However, if antibiotics are used indiscriminately, the pathogen could become more resistant, resulting in antibiotic resistance. By using this scoring system, we are able to identify high-risk individuals. If new infections are uncontrollable for these high-risk CR-GNB carrier patients, it seems a better option to use empirical anti-CR-GNB antibiotics for the treatment of these high-risk patients.

This study had several limitations. First, our data, acquired from 2020 and 2022, are a partial representation of people with GEDs. This cohort, from a tertiary hospital in Zhejiang province, was not representative of all Chinese patients in GED. Additionally, not all potential factors affecting secondary CR-GNB BSI from colonization were considered in the risk factor analysis. Several factors that could contribute to CR-GNB BSI risk were not fully disclosed, including occupations and other conditions. Third, although a universal gold standard method has not been yet described in making the definitive diagnosis of acute cholangitis, Charcot’s triad, which was used by clinicians in our study, is not as sensitive as the Tokyo 2018 criteria in the diagnosis of acute cholangitis [32]. Therefore, it may miss many cases of acute cholangitis. The fourth issue is that, although we extensively validated the robustness of our nomogram using bootstrap testing, no external validation was conducted. It is unclear whether the results are generalizable to other GED populations in different regions and countries. There is a need for external evaluation in a wider population of patients.

## 5. Conclusions

In conclusion, on the basis of logistic regression analysis, a predictive nomogram that included six variables was established to assess secondary CR-GNB BSI risk from colonization in GED patients. As demonstrated by internal validation, the nomogram performed well. Using the predictive nomogram to score, we might thus target risk factors for control. By using the nomogram, patients with secondary CR-GNB BSI can be identified at an early stage. To reduce the mortality of CR-GNB BSI, empirical anti-CR-GNB antibiotics can be prescribed in a timely fashion in a selective manner. The nomogram needs further external validation to be optimized and improved.

## Figures and Tables

**Table 1:** Demographic and clinical characteristics of patients of both case and control groups.

Variables	Controls (CR-GNB Rectal Carriers Who Did Not Develop Secondary BSI) *n* = 90 (%)		Cases (CR-GNB Rectal Carriers Who Developed Secondary BSI) *n* = 90 (%)		Z/X2	*p* Value
	*n*	%	*n*	%		
Age, year	65.9 (62.1–69.7) (62.1–69.7) 69.7		64.3 (60.9–67.6)		−0.58	0.563
Gender					0.097	0.756
Male	57	63%	59	66%		
Female	33	37%	31	34%		
Ward					1.133	0.568
Ward 1	52	58%	45	50%		
Ward 2	22	24%	27	30%		
Ward 3	16	18%	18	20%		
CR-GNB Isolates					16.585	0.002
*Klebsiella pnenmoniae*	36	40%	54	60%		
*Escherichia coli*	26	29%	19	21%		
*Enterobacter cloacae*	14	15%	4	5%		
*Citrobacter freundii*	10	11%	3	3%		
*Pseudomonas aeruginosa*	4	5%	10	11%		
ECOG Scores					24.533	<0.001
ECOG scores 0	6	7%	1	1%		
ECOG scores 1	15	17%	4	4%		
ECOG scores 2	27	30%	12	13%		
ECOG scores 3	33	37%	53	59%		
ECOG scores 4	9	10%	20	22%		
Past History						
long-term stay in healthcare facility within 1 year	24	2%	50	56%	3.107	0.078
ICU admission history within 1 year	10	11%	12	13%	0.207	0.650
blood-stream infection history within 1 year	9	10%	8	9%	0.065	0.799
Provenance of Patient at Admission						
Transfer from another healthcare facility	38	42%	42	47%	0.360	0.550
Diseases						
gastrointestinal bleeding	12	13%	9	10%	0.485	0.486
inflammatory bowel disease	2	2%	2	2%	0.000	1.000
severe acute pancreatitis	10	11%	30	33%	12.857	<0.001
acute cholangitis	20	22%	25	28%	0.741	0.391
cirrhosis	19	21%	22	24%	0.284	0.595
gastrointestinal obstruction	10	11%	7	8%	0.585	0.446
gastrointestinal cancer	11	12%	13	14%	0.192	0.662
diabetes	20	22%	33	37%	4.519	0.034
cardial vascular disease	40	44%	35	39%	0.571	0.451
cerebral vascular disease	9	10%	12	13%	0.485	0.487
pulmonary disease	15	17%	15	17%	0.000	1.000
uremia	2	2%	2	2%	0.000	1.000
neutropenia	6	7%	24	27%	12.960	<0.001
Admission Duration						
Length of stay from CR-GNB screen to outcome	12 (12.1–15.1)		13 (14.3–18.3)			0.092
Long-term stay in hospital	39	43%	50	56%	15.512	<0.001
Surgical History						
GI divert	8	9%	9	10%	0.065	0.799
Endoscopy Interventions after Survey						
gastroscopy	25	28%	23	26%	0.114	0.737
colonoscopy	7	8%	5	8%	0.357	0.551
ERCP	29	32%	33	55%	0.394	0.532
Other Interventions after Survey						
enbd	25	28%	28	47%	0.241	0.625
small bowel feeding tube	14	16%	3	3%	7.860	0.005
small bowel decompression tube	6	7%	4	7%	0.106	0.745
colon decompression tube	5	6%	5	8%	0.00	1.000
deep venous catheter	22	24%	25	42%	0.259	0.612
dialysis	2	2%	2	3%	0.000	1.000
cholecystostomy	7	8%	10	17%	0.585	0.446
PTCD	5	6%	8	13%	0.746	0.389
parenteral nutrition above 3 days	10	11%	32	36%	15.031	<0.001
Drugs after Survey						
chemotherapy	8	9%	14	16%	1.864	0.173
steroid usage above 5 days	5	4%	7	16%	0.357	0.551
Antibiotics after Survey						
β-lactam-β-lactamase inhibitor	30	33%	36	40%	0.861	0.355
cephalosporins	53	59%	62	69%	1.951	0.164
quinolone	23	26%	21	23%	0.120	0.729
carbapenem	28	31%	37	41%	1.951	0.164
poly antibiotics	46	51%	51	57%	0.559	0.456

Footnotes: CR-GNB = carbapenem-resistant Gram-negative bacteria, ECOG = Eastern cooperative oncology group score, *n* = number of patients, *p* = test significance, ERCP = endoscopic retrograde cholangiopancreatography, PTCD = percutaneous transhepatic cholangial drainage, ICU = intensive care unit.

**Table 2:** Univariate and multivariate analysis of risk factors for secondary CR-GNB BSI in CR-GNB intestinal carrier patients in gastroenterology department.

Variables	Univariate Analysis		Multivariate Analysis	
	OR (95% CI)	*p* Value	OR (95% CI)	*p* Value
Age, year	/	0.521	N/A	N/A
Gender	1.10 (0.60–2.03)	0.756	N/A	N/A
Ward	1.18 (0.81–1.72)	0.389	N/A	N/A
CR-GNB isolates	0.84 (0.67–1.07)	0.158	N/A	N/A
*Klebsiella pnenmoniae*	2.25 (1.24–4.09)	0.008	1.08 (0.45–2.60)	0.861
*Escherichia coli*	0.66 (0.33–1.30)	0.230	N/A	N/A
*Enterobacter cloacae*	0.25 (0.08–0.80)	0.019	N/A	N/A
*Citrobacter freundii*	0.28 (0.07–1.04)	0.057	0.21 (0.04–1.23)	0.084
*Pseudomonas aeruginosa*	2.69 (0.81–8.91)	0.106	N/A	N/A
ECOG scores	2.24 (1.56–3.21)	<0.001	5.68 (2.96–10.90)	<0.001
Past History				
long-term stay in healthcare facility within 1 year	1.769 (0.94–3.35)	0.079	N/A	N/A
ICU admission history within 1 year	1.23 (0.50–3.01)	0.649	N/A	N/A
blood stream infection history within 1 year	0.88 (0.32–2.39)	0.799	N/A	N/A
Provenance of Patient at Admission				
Transfer from another healthcare facility	1.20 (0.67–2.16)	0.549	N/A	N/A
Diseases				
gastrointestinal bleeding	0.72 (0.29–1.81)	0.487	N/A	N/A
inflammatory bowel disease	1.00 (0.14–7.26)	1.000	N/A	N/A
severe acute pancreatitis	4.00 (1.82–8.81)	0.001	6.32 (2.02–19.81)	0.002
acute cholangitis	1.35 (0.68–2.65)	0.390	N/A	N/A
cirrhosis	1.21 (0.60–2.43)	0.594	N/A	N/A
obstruction	0.68 (0.25–1.86)	0.447	N/A	N/A
gastrointestinal cancer	1.21 (0.51–2.87)	0.661	N/A	N/A
diabetes	2.03 (1.05–3.91)	0.035	4.02 (1.40–11.50)	0.01
cardial vascular disease	0.80 (0.44–1.44)	0.450	N/A	N/A
cerebral vascular disease	1.39 (0.55–3.47)	0.487	N/A	N/A
pulmonary disease	1.00 (0.46–2.19)	1.000	N/A	N/A
uremia	1.00 (0.14–7.26)	1.000	N/A	N/A
neutropenia	5.09 (1.97–13.18)	0.001	4.77 (1.4–15.76)	0.01
Admission Duration				
Long-term stay in hospital (days in hospital > 12 days)	3.44 (1.84–6.43)	<0.001	5.32 (2.25–12.54)	<0.001
Surgery History				
GI divert	1.14 (0.42–3.10)	0.799	N/A	N/A
Endoscopy Interventions after Survey				
gastroscopy	0.89 (0.46–1.73)	0.736	N/A	N/A
colonoscopy	0.70 (0.21–2.29)	0.552	N/A	N/A
ERCP	1.22 (0.66–2.25)	0.531	N/A	N/A
Other Interventions after Survey				
enbd	1.17 (0.62–2.23)	0.624	N/A	N/A
small bowel feeding tube	0.19 (0.05–0.68)	0.011	0.12 (0.02–0.70)	0.019
small bowel decompression tube	0.65 (0.18–2.39)	0.518	N/A	N/A
colon decompression tube	1.00 (0.28–3.58)	1.000	N/A	N/A
deep venous catheter	1.19 (0.61–2.31)	0.611	N/A	N/A
dialysis	1.00 (0.14–7.26)	1.000	N/A	N/A
cholecystostomy	1.48 (0.54–4.08)	0.447	N/A	N/A
PTCD	1.66 (0.52–5.28)	0.392	N/A	N/A
parenteral nutrition above 3 days	4.41 (2.01–9.69)	<0.001	9.01 (2.77–29.36)	<0.001
Drugs Usage after Survey				
chemotherapy	1.888 (0.75–4.75)	0.177	N/A	N/A
steroid usage above 5 days	1.43 (0.44–4.70)	0.552	N/A	N/A
Antibiotics Usage after Survey				
β-lactam-β-lactamase inhibitor	1.33 (0.73–2.45)	0.354	N/A	N/A
cephalosporins	1.55 (0.84–2.85)	0.164	N/A	N/A
quinolone	0.89 (0.45–1.75)	0.729	N/A	N/A
carbapenem	1.55 (0.84–2.85)	0.164	N/A	N/A
poly antibiotics	1.25 (0.70–2.25)	0.455	N/A	N/A

CR-GNB = carbapenem-resistant Gram-negative bacteria, ECOG = Eastern cooperative oncology group score, OR = odds ratio, 95% CI = confidence interval, *p* = test significance, ERCP = endoscopic retrograde cholangiopancreatography, PTCD = percutaneous transhepatic cholangial drainage, ICU = intensive care unit, N/A = not applicable.

## Data Availability

The data are available upon reasonable request to the corresponding authors after approval from the Ethics Committee of Hangzhou First People’s Hospital.
